# Experimental GPR datasets to characterize multi-layered pavement structures–Tack coat evaluation using hybridization method

**DOI:** 10.1016/j.dib.2025.112009

**Published:** 2025-08-27

**Authors:** Grégory Andreoli, Amine Ihamouten, David Souriou, David Guilbert, Mai Lan Nguyen, Xavier Dérobert

**Affiliations:** a Gustave Eiffel University, MAST/EMGCU – Champs-sur-Marne, F-13300 Salon-de-Provence, France; b Gustave Eiffel University, MAST/LAMES – Nantes, F-44344 Bouguenais, France; c FI-NDT– Nantes, F-44344 Bouguenais, France; d Gustave Eiffel University, DGS/DGDIN/DAR – Nantes, F-44344 Bouguenais, France; e Gustave Eiffel University, GeoEND – Nantes, F-44344 Bouguenais, France

**Keywords:** Interface, Radar, Electromagnetism, Machine learning

## Abstract

The use of non-destructive testing methods (NDT) on newly constructed pavements serves both to verify the proper design of the delivered infrastructure and to support its ongoing monitoring. However, once construction is complete, conventional GPR methods are no longer capable of accurately assessing the thinnest layers, millimeter-scale variations, or the precise amount of bituminous emulsion required to ensure proper bonding at the interfaces. To support this research, a database was created using an impulse Ground Penerating Radar (GPR) with multiple central frequencies on controlled and multi-layered pavement structures according to current French standards [1,2]. Variations in materials and geometries were applied to the two upper courses (wearing course and binder course layers) and tack coat emulsion quantity at the interface. The objective is to obtain a representative database for experimental validation, enabling the characterization of the tack coat located between the two layers that form the surface layer [3]. A recent research study conducted on synthetic data led to a comprehensive parametric analysis [4], validating the methodology of a hybrid Machine Learning/FWI data processing approach to enhance performance and reduce computation times [5]. The current challenge is to distinguish between areas with and without tack coat, as well as to detect subtle geometric variations, using an impulse GPR system equipped with ground-coupled bowtie antennas on a pavement structure fabricated under controlled conditions. The objective is to classify the acquired data and map the results of the Support Vector Machine (SVM) model as an electromagnetic image.

Specifications table

**Table:** 

Subject	Engineering & Materials science
Specific subject area	Experimental Tack coat Characterization with radar methods.
Type of data	Raw Data (*.DZT), Filtered Data (*.DZX): GSSI format Table (*.csv): conversion of raw DZT file.l
Data collection	This database is created using GSSI SIR®4000 device with two different antennas (350 MHz and 2.6 GHz) on multi-layered pavement structures in accordance with current French standards [1,2]. Variations in geometrical parameters were applied to each section to have many realistic configurations.
Data source location	Experimental GPR dataset on the fatigue carouse of Gustave Eiffel University (Nantes Campus) Location: 47°09′10.9"N 1°38′34.9"Wl
Data accessibility	Repository name: Dataverse: recherche.data.gouv.fr Data identification number: 10.57745/OXOEZM Direct URL to data: https://doi.org/10.57745/OXOEZM Version: - 1.1: ReadMe_OXOEZM_2025_en (2025-08-12) – English version - 1.1: ReadMe_OXOEZM_2025_fr (2025-08-12) – French version
Related research article	*The dataset is relative to the article DOI:*https://doi.org/10.1016/j.treng.2025.100339 *Named: Hybrid ML/FWI method using GPR data to evaluate the tack coat characteristics in pavements: Experimental validation*

## 1 Value of the Data

• The datasets consist of two-dimensional graphical matrices (distance, time), representing experimental electromagnetic reflections (B-scans) acquired on a full-scale pavement structure with controlled material formulation and geometry in a laboratory setting. The geometry varies across the wearing and binder courses of the pavement. A tack coat is applied over part of the structure. All acquisition parameters are provided for each dataset. Additionally, the dielectric permittivity of the surface layer is included, enabling its use as a relevant physical *a priori* for hybrid data processing approaches combining Machine Learning and Full Waveform Inversion (ML/FWI).
• Generating experimental data that reliably represents a well-controlled bonding condition at subsurface interfaces—on a structure with known geometry and controlled aging—is both rare and difficult to achieve. Yet, this type of data is crucial, particularly as input for developing machine learning or deep learning models;
• In addition to output files in both raw and filtered format, the tabular form facilitates reading of the database without any dedicated tools;
• These experimental datasets, acquired using a GSSI system, are shared with the broader scientific and professional pavement community, particularly those working with GPR technologies. They are intended to support the detection and characterization of the tack coat interface between the wearing course and the binder course, in alignment with current French standards.

## 2 Background

Surface pavement deterioration and the emergence of visible defects are often indicative of underlying subsurface anomalies. Phenomena such as delamination, stripping of the wearing course, and pothole formation can result from a combination of factors, including the cumulative effects of climate change, increased traffic loading, and inadequate application of the tack coat [6,7]. This ultra-thin layer, located at the interface between the wearing and binder courses, plays a critical role in ensuring adhesion between layers, thereby mitigating shear stresses induced by traffic loads.

For nearly fifty years, Gustave Eiffel University (Nantes campus) has used a high-capacity full-scale accelerated pavement testing device known as the *Fatigue Carousel*, designed to simulate controlled long-term aging of transport infrastructure [8]. Pavement sections used in the device are constructed with carefully monitored formulations and implementation procedures. In this study, impulse radar data were collected using antennas with central frequencies of 350 MHz (HyperStacking) and 2.6 GHz, both at the initial (as-built) state and after 50,000 loading cycles (equivalent to heavy truck traffic traveling at 90 km/h).

The dataset collected in this research work comprises radar measurements reflecting variations in central frequencies, structural geometry, mean texture depth, binder content, interlayer bonding quality and aging structure. These data serve to support the validation of inverse modeling methods aimed at characterizing the subsurface tack coat layer within a multilayer pavement structure.

## 3 Data Description

Global database includes raw A-scans data collected for all the profiles outlined in the previous section, using GSSI device. The first-level folders are dedicated to each center frequency antennas: 350 MHz and 2.6 GHz. Each subfolder indicating the frequency in MHz at the beginning. The data structure tree is described as follows:

• Experimental_GPR_Database
• /350MHz
  ○ /350_T0
    ■ /RAW
      ■ 350_T0_Profil_name_Section_name.DZT
      ■ 350_T0_Profil_name_Section_name.DZX
      ■ 350_T0_Air.DZT
      ■ 350_T0_ Air.DZX
      ■ /CSV
      ■ 350_T0_Profil_name_Section_name.CSV
      ■ 350_T0_Air.CSV
    ■ /TXT
      ■ 350_T0_Profil_name_Section_name.TXT
      ■ 350_T0_Air.TXT
  ○ /350_T1
    ■ /RAW
      ■ 350_T1_Profil_name_Section_name.DZT
      ■ 350_T1_Profil_name_Section_name.DZX
      ■ 350_T1_Air.DZT
      ■ 350_T1_ Air.DZX
    ■ /CSV
      ■ 350_T1_Profil_name_Section_name.CSV
      ■ 350_T1_Air.CSV
    ■ /TXT
      ■ 350_T1_Profil_name_Section_name.TXT
      ■ 350_T1_Air.TXT
• /2600MHz
  ○ /2600_T0
    ■ /RAW
      ■ 2600_T0_Profil_name_Section_name.DZT
      ■ 2600_T0_Profil_name_Section_name.DZX
      ■ 2600_T0_Air.DZT
      ■ 2600_T0_ Air.DZX
    ■ /CSV
      ■ 2600_T0_Profil_name_Section_name.CSV
      ■ 2600_T0_Air.CSV
    ■ /TXT
      ■ 2600_T0_Profil_name_Section_name.TXT
      ■ 2600_T0_Air.TXT
  ○ /2600_T1
    ■ /RAW
      ■ 2600_T1_Profil_name_Section_name.DZT
      ■ 2600_T1_Profil_name_Section_name.DZX
      ■ 2600_T1_Air.DZT
      ■ 2600_T1_ Air.DZX
    ■ /CSV
      ■ 2600_T1_Profil_name_Section_name.CSV
      ■ 2600_T1_Air.CSV
    ■ /TXT
      ■ 2600_T1_Profil_name_Section_name.TXT
      ■ 2600_T1_Air.TXT

**Fig. 1:**
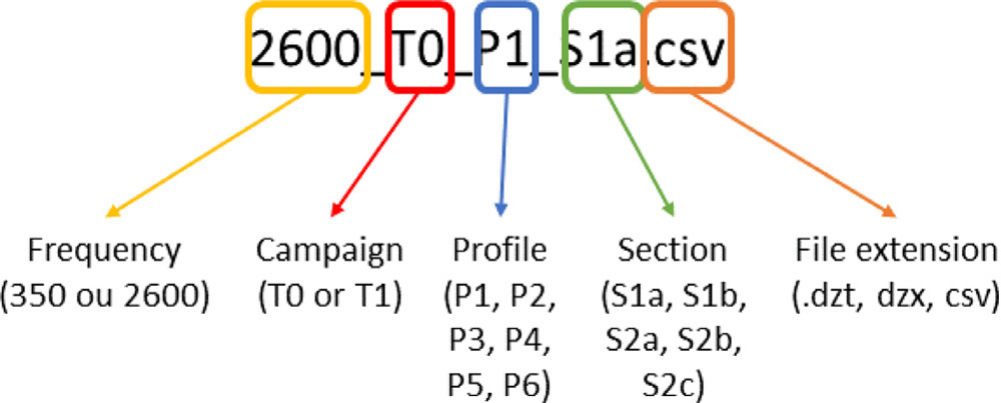
Example file description.

At the second level, the folders are organized based on used frequency and the campaign test (T0: initial state; T1: after 50,000 loading cycles). At the third level, the folders include /RAW (containing all GSSI raw data *.dzt and *.dzx), /CSV and /TXT (converted files from *.dzt to *.csv format). For each measurement campaign and each frequency, an air shot was performed as a reference (*_Air.*). The data files are named according to the specified nomenclature (Fig. 1):

• The *.csv file derived from the *.dzt file contains a number of rows corresponding to the number of A-scans and a number of columns corresponding to the amplitude values of each discrete A-scan.

## 4 Experimental Design, Materials and Methods

**Fig. 2:**
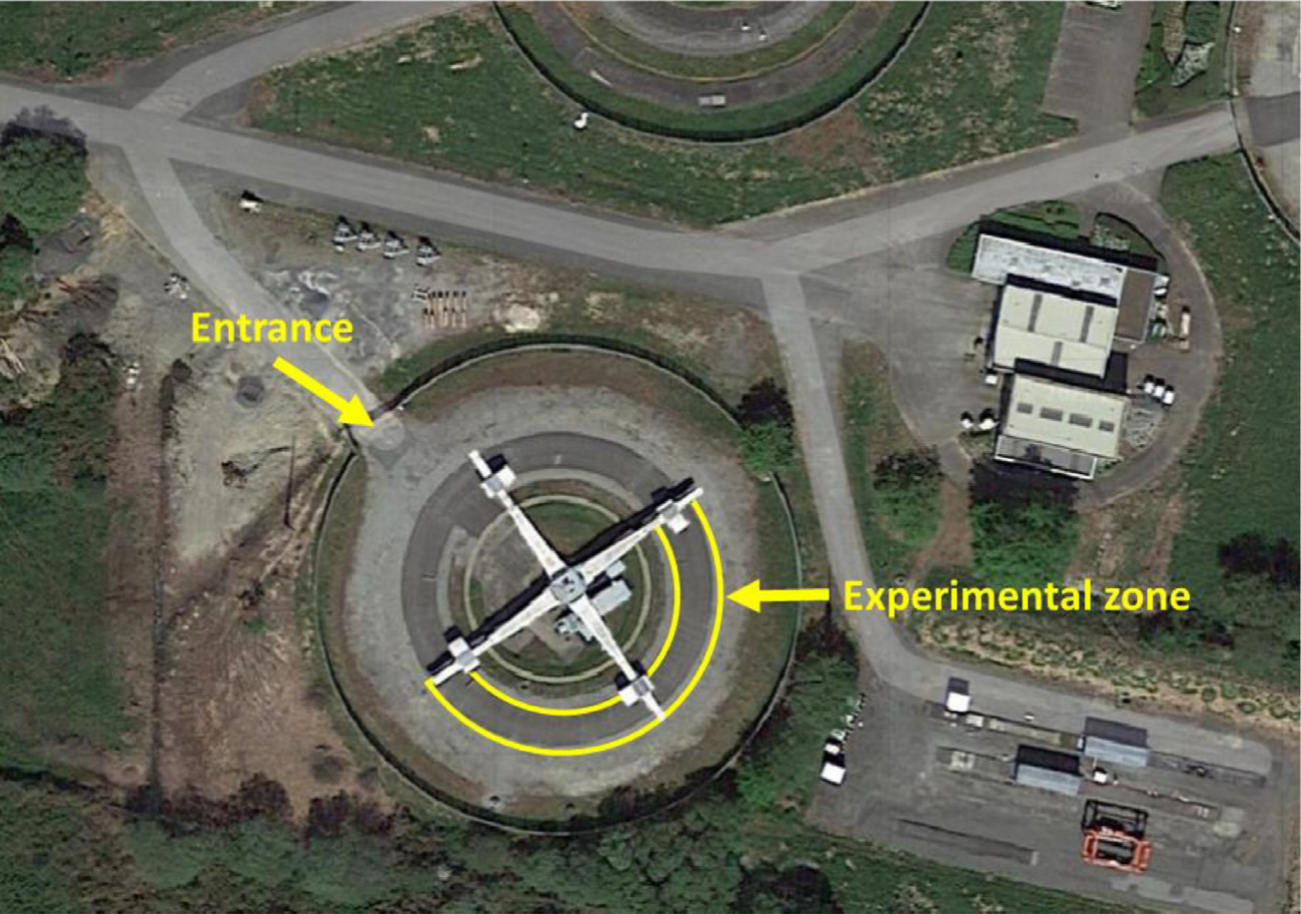
Fatigue carousel at Gustave Eiffel University in Nantes (France).

The fatigue carousel (Fig. 2) at Gustave Eiffel University in Nantes is a road traffic simulator designed to accelerate pavement degradation. It is equipped with four cross-arranged arms that apply significant loads (up to 65 kN on a single wheel) in tandem or tridem configurations. The rotation speed is adjustable and can reach up to 90 km/h. Thus, two measurement campaigns were conducted: the first (T0) corresponds to the initial state of the pavement (before the fatigue carousel loading), while the second (T1) represents the pavement's aging after 50,000 loading cycles.

**Fig. 3:**
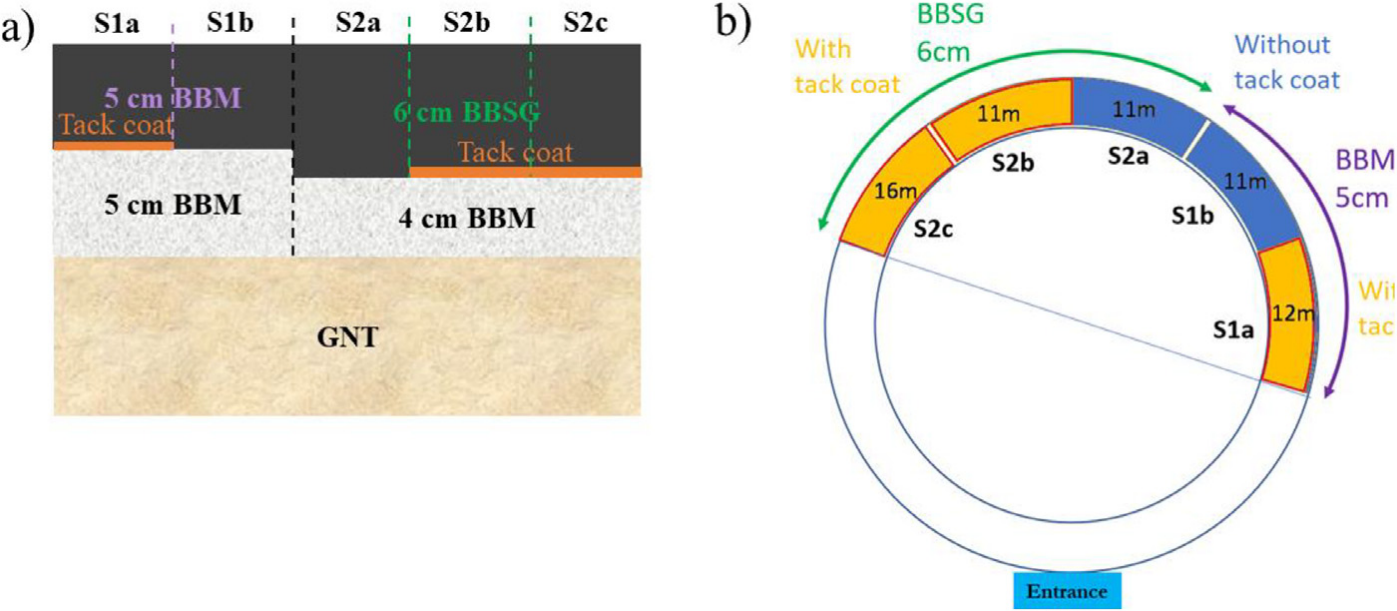
BINARY Structure on the fatigue carousel: a) Various tri-layers structure; b) Section on the carousel [10].

A pavement structure is manufactured for the ANR BINARY project [9] on a circular test track measuring 60 meters in length and 3.5 meters in width. It’s a new BBM (French acronym for *Béton Bitumineux Mince* corresponding to Thin Bituminous Concrete wearing course) on a BBM old binder course or a new BBSG (French acronym for *Béton Bitumineux Semi-Grenu* corresponding to Semi-Coarse Bituminous Concrete) on the same old BBM Binder course. Notably, an existing surface layer was partially milled to accommodate the new structure in different sections (Fig. 3a). The test area includes five sections with varying surface layer geometries, incorporating two different bituminous emulsion dosages (Fig. 3b). Sections S2b and S2c are identical. The selected aggregates were sourced from Carrières et Matériaux du Grand Ouest (CMGO) in Rouans, France. Their mineralogical composition is primarily gneiss (a metamorphic rock) with a dielectric permittivity ranging from 4.9 to 5.5. The structural characteristics of the pavement are presented in Table 1. Sections S1a, S2b, and S2c include a bituminous emulsion applied at a dosage of 300 g/m² between the wearing and binder courses.

**Table 1:** Material and structure characteristics.

Section (see Fig. 2b)	Distance (m)	Wearing course (+ class)	Thickness (cm)	Graded aggregate (mm)	Binder content (%)	Air-void content (%)	Mean Depth Texture (mm)	Tack coat emulsion quantity (g/m²)	Binder course	Thickness (cm)
S1a	12	BBM A	5	0/10	5,48	6,5	1,25	300	BBM	5
S1b	11	BBM A	5	0/10	5,48	6,5	1,25	0	BBM	5
S2a	11	BBSG 3	6	0/10	5,63	6,5	0,83	0	BBM	4
S2b	11	BBSG 3	6	0/10	5,63	6,5	0,83	300	BBM	4
S2c	16	BBSG 3	6	0/10	5,63	6,5	0,83	300	BBM	4

The extraction of quantitative information about the controlled structure allows, using an impulse GPR device at two different central frequencies (350 MHz and 2.6 GHz), to characterize the geometry and the presence of the tack coat in the subsurface, as well as the different fatigue states of the structure.

**Fig. 4:**
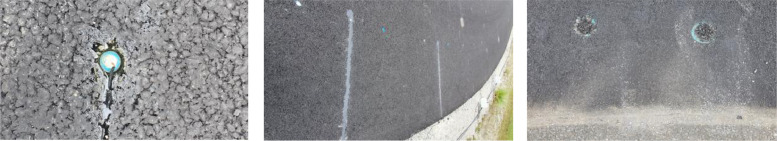
Instrumentation of the surveyed area that may affect GPR radar measurements; a) Geophones; b) Location of optical cables; c) Core samples refilled with cold-pour asphalt.

For the purpose of other experiments, it is important to note that the controlled pavement structure was heavily instrumented. Surface-mounted geophones can be seen at the center of the driving lane (Fig. 4a), while buried optical cables cross the lane transversely (Fig. 4b). Additionally, horizontal sensors and fiber optic sensor plates are present. Moreover, for a complementary gamma densitometry study, two 100 mm diameter core samples were taken per section near the inner edge (Fig. 4c).

The device used in the experimental campaign was the SIR®4000 GPR system [11], manufactured by GSSI, in combination with 350 MHz HyperStacking antennas [12] and 2.6 GHz impulse ground-coupled bowtie antennas [13].

**Fig. 5:**
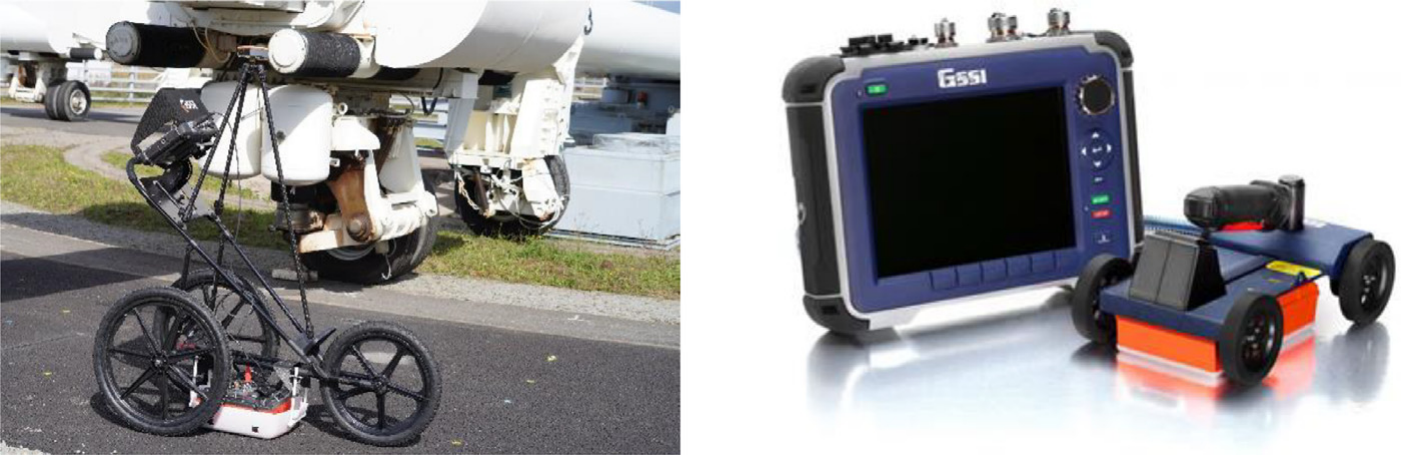
GPR device: a) SIR®4000 with 350 MHz HyperStacking antennas on stroller; b) SIR®4000 with 2.6 GHz antennas.
• For 2.6 GHz antennas (Fig. 5b): 2045 sampling A-scans, a time range of 5 ns and a spatial sampling of 2 mm, Filter FIR = off, Filter IIR = [0.4, 5] GHz.

**Fig. 6:**
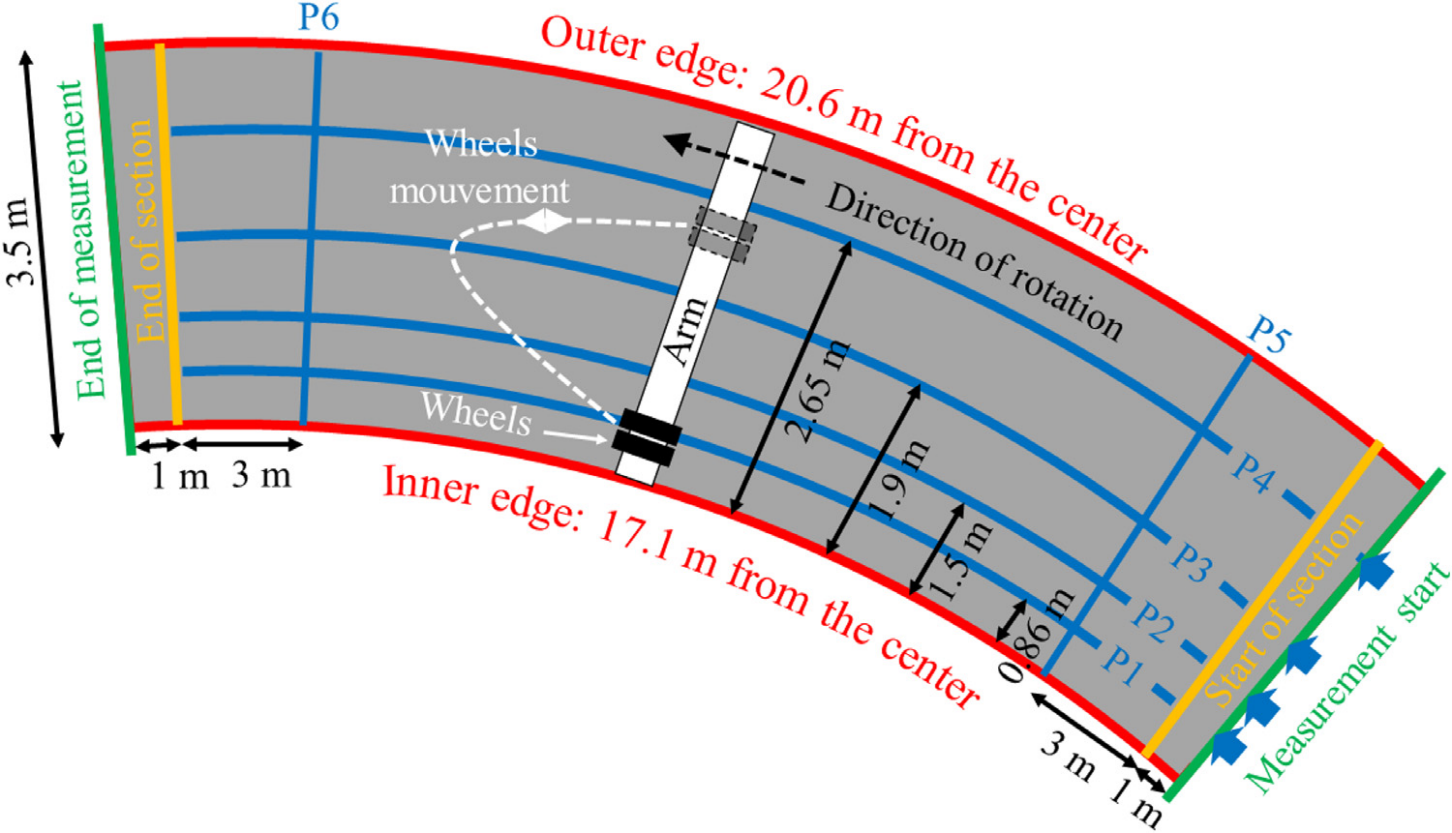
Experimental protocol and measurement profiles [10].

The setup parameters were as follows:

• For the 350 MHz antennas (Fig. 5a): 2045 sampling A-scans, a time range of 25 ns and as spatial sampling of 2 mm, Filter FIR = off, Filter IIR = [0.4, 5] GHz;

The surveyed area is shaped like an arc, 3.5 meters wide, with a variable length depending on the section (see Table 1). Based on the different structural geometries and tack coat emulsion quantity, as well as the constraints imposed by the existing instrumentation and the wheel positions during the fatigue carousel loading (with the wheels moving along the arm following a Gaussian distribution while rotating), different survey profiles were considered (Fig. 6).

**Weather conditions at T0:**

• Sunny (with light rain 2 days before),
• Location: Experimental GPR dataset on the fatigue carouse of Gustave Eiffel University (Nantes Campus) - 47°09′10.9"N 1°38′34.9"W,
• Date of experiment: Wednesday 29 September 2021,
• Temperature during measurements: 18°C,
• Measurements started with 350 MHz antennas: 12:42 a.m.,
• Measurements ended with 350 MHz antennas: 2:07 p.m.,
• Measurements started with 2.6 GHz antennas: 2:23 p.m.,
• Measurements ended with 2.6 GHz antennas: 3:46 p.m.

**Weather conditions at T1:**

• Sunny (with light fog in the morning),
• Location: Experimental GPR dataset on the fatigue carouse of Gustave Eiffel University (Nantes Campus) - 47°09′10.9"N 1°38′34.9"W,
• Date of experiment: Monday 21 February 2022,
• Temperature during measurements: 7°C (in the morning) to 15°C (in the afternoon),
• Measurements started with 350 MHz antennas: 8:57 a.m.,
• Measurements ended with 350 MHz antennas: 1:25 p.m.,
• Measurements started with 2.6 GHz antennas: 2:12 p.m.,
• Measurements ended with 2.6 GHz antennas: 3:05 p.m.

The survey profiles are defined as describe in Table 2.

**Table 2:** Profiles description.

Profile name	Direction	Distance	Distance from the center	Comments
P1	Longitudinal	0.86 m from the inner circle	17.9 m	Minimal wheel loading from the carousel
P2	Longitudinal	1.5 m from the inner circle	18.6 m	Moderately loaded zone
P3	Longitudinal	1.9 m from the inner circle	19 m (reference)	Wheel-loaded zone
P4	Longitudinal	2.65 m from the inner circle	19.75 m	Non-loaded zone, included optical cable
P5	Transverse	3 m after the beginning of the section	From 20.5 to 17 m	Avoid instrumentation (see Fig. 3)
P6	Transverse	3 m before the end of the section	From 20.5 to 17 m	Avoid instrumentation (see Fig. 3)

The starting point for longitudinal measurements in each section begins 1 meter before and ends 1 meter after the section. This protocol ensures that each section to be evaluated is properly framed and helps compensate for any potential misalignment between the theoretical and actual start/end points of the emulsion application. It is important to note, in the specific case of the measurement 350_T0_P3_S2a.* (naming convention explained below), the measurement was stopped 3 meters after the section instead of 1 meter. The starting point for transversal measurements in each section begins **at the outer edge line of the track** and ends **at the inner edge line.**

Based on various collected data, it is possible to identify geometric variations in the different layers of the infrastructure, as well as to build annotated databases for classifying the bonding condition at the interface.

**Fig. 7:**
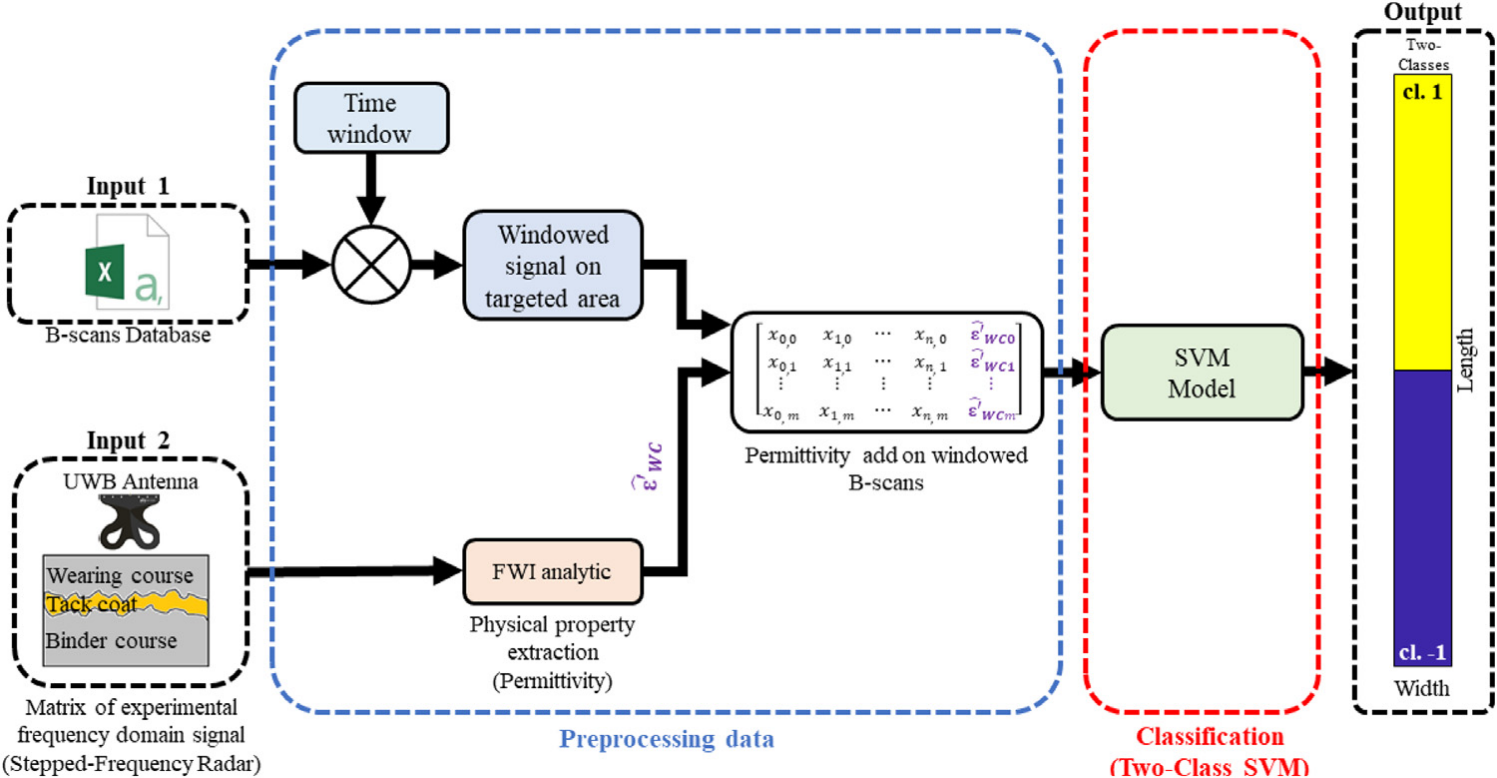
Flowchart illustrating the B–scans processing hybrid method.

Once the radar data have been collected using the method described above, they can be organized into a matrix format to discriminate between pavement sections with or without tack coats. Moreover, thanks to the detailed description of the data format, the dataset can be extended with new measurements, thereby improving the robustness of the classification when applied to previously unseen pavements (Fig. 7 – Input 1).

Building on the methodology outlined in the reference article [10], the use of a calibrated UWB antenna with a vector network analyzer enables the extraction of the dielectric permittivity of the wearing course using the Full Waveform Inversion (FWI) method (Fig. 7 – Input 2).

Combining the dielectric permittivity of the wearing course with the radar data matrix allows for a hybrid AI/FWI data processing approach. This methodology has proven effective in several research studies [[1], [2], [3], [4], [5]], demonstrating improved performance in machine learning-based classification and estimation tasks thanks to the incorporation of structural *a priori* information.

For the pavement structure investigated in this study, a dielectric permittivity of approximately ε_WC_ ≈ 4.4 was observed for the wearing course, averaged across all sections. This value is then applied to the entire dataset during the pre-processing phase (Fig. 7).

**Fig. 8:**
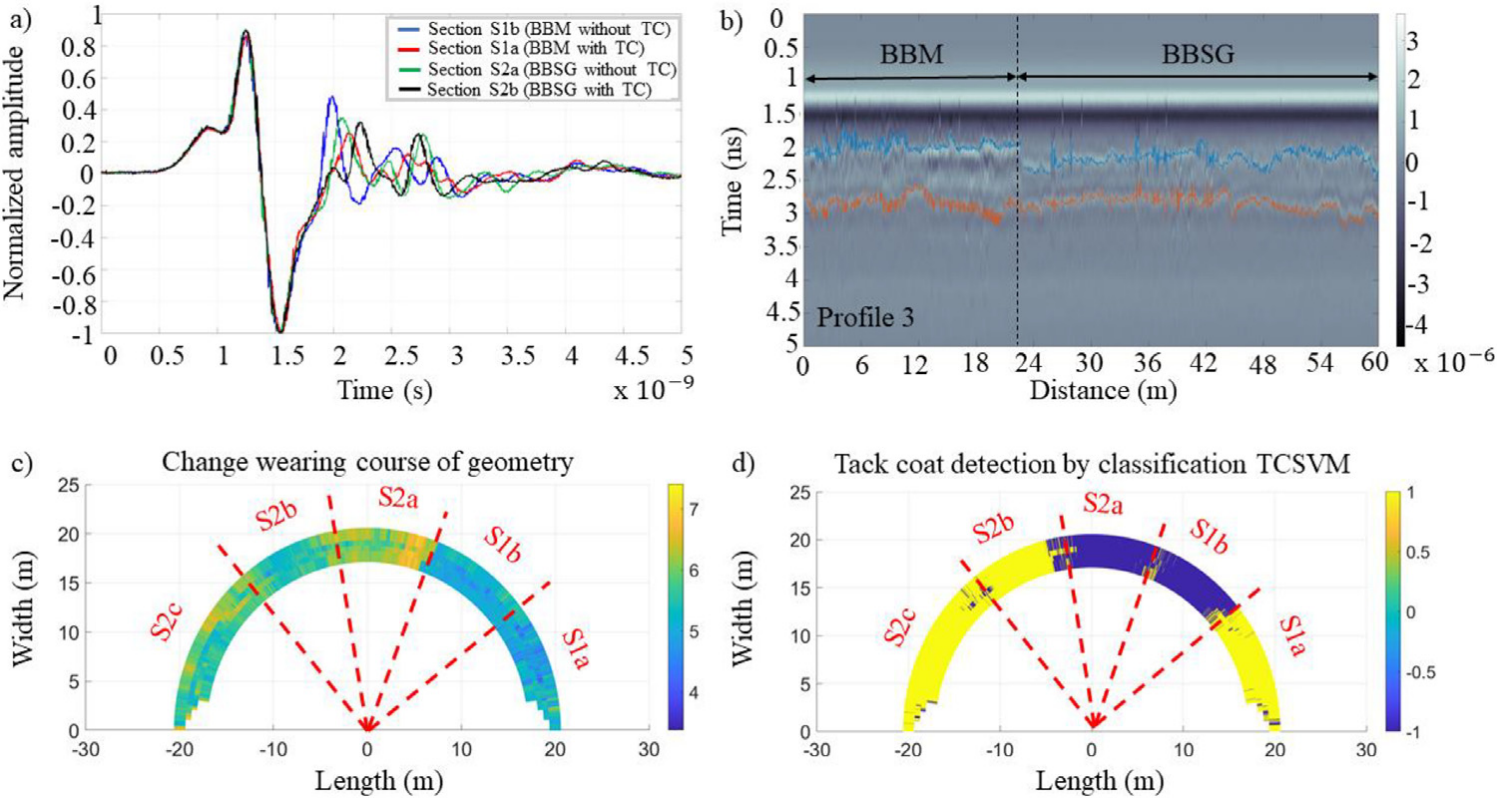
Results from the fatigue carousel [10]: a) Example of A-scans from each section on Profile P3; b) B-scan on Profile P3; c) Circular projection of the surface layer geometry; d) Radar data inversion results for TCSVM classification at T0.

The set of A-scans (Fig. 8a) forming the database described in this research demonstrates various potential applications, such as estimating the wearing course geometry (Fig. 8b and c) and classifying areas with or without tack coat (Fig. 8d).

These experimental databases, gathered from the fatigue carousel at Gustave Eiffel University (Nantes, France), are openly shared with the scientific and professional pavement community, with a particular focus on GPR applications. They serve as a valuable resource for detecting and characterizing the tack coat interface between the wearing course and the binder course, in alignment with current standards.

## Limitations

This experiment enabled the discrimination between two tack coat conditions. A greater variation in application rates could have allowed for the use of multi-class SVM or SVR approaches to achieve a more comprehensive classification.

## Ethics Statement

The present work did not involve the use of human subjects, animal experiments, or data collected from social media platforms.

## Credit Author Statement

**Grégory Andreoli:** Conceptualization, Methodology, Investigation, write original draft; **Amine Ihamouten:** Conceptualization, Supervision, Writing and Review & Editing, Validation; **David Souriou:** Investigation; **David Guilbert:** Investigation; **Mai Lan Nguyen:** Resources, Investigation; **Xavier Dérobert:** Supervision, Review & Editing, Validation.

## Data Availability

DataverseExperimental GPR database (Inverse approach for pavement tack coat characterization) (Reference data).
